# Gold and Clarke Questionnaires Identify Different People With Insulin‐Treated Diabetes and Impaired Awareness of Hypoglycaemia in Almost 60% of Cases: A Post Hoc Analysis From the Hypo‐METRICS Study

**DOI:** 10.1111/dom.70869

**Published:** 2026-05-11

**Authors:** Vaios Koutroukas, Jonah Thomas, Gilberte Martine‐Edith, Aikaterina Tziannou, Patrick Divilly, Uffe Søholm, Claire L. Meek, Simon Heller, Julia K. Mader, Ulrik Pedersen‐Bjergaard, Bastiaan E. de Galan, Eric Renard, Mark L. Evans, Rory J. McCrimmon, Jane Speight, Frans Pouwer, Stephanie A. Amiel, Pratik Choudhary, the Hypo‐RESOLVE consortium

**Affiliations:** ^1^ Diabetes Research Centre, University of Leicester, College of Life Sciences Leicester UK; ^2^ Leicester Diabetes Centre, Leicester General Hospital Leicester UK; ^3^ St. Vincent's University Hospital, Dublin, University College Dublin Dublin Ireland; ^4^ School of Psychology, Deakin University Melbourne Australia; ^5^ The Australian Centre for Behavioural Research in Diabetes, Diabetes Victoria Melbourne Victoria Australia; ^6^ Division of Clinical Medicine School of Medicine and Population Health, University of Sheffield Sheffield UK; ^7^ Division of Endocrinology and Diabetology, Department of Internal Medicine Medical University of Graz Graz Austria; ^8^ Department of Clinical Medicine, Faculty of Health and Medical Sciences University of Copenhagen Copenhagen Denmark; ^9^ Department of Endocrinology and Nephrology North Zealand Hospital Hillerød Denmark; ^10^ Department of Internal Medicine Radboud University Medical Center Nijmegen the Netherlands; ^11^ Department of Internal Medicine Maastricht University Medical Center+ Maastricht the Netherlands; ^12^ CARIM Cardiovascular Research Institute Maastricht, Maastricht University Maastricht the Netherlands; ^13^ Department of Endocrinology, Diabetes, Nutrition, Montpellier University Hospital Montpellier France; ^14^ Institute of Functional Genomics, University of Montpellier Montpellier France; ^15^ Wolfson Diabetes and Endocrine Clinic Cambridge University Hospitals NHS Foundation Trust Cambridge UK; ^16^ Institute of Metabolic Science, University of Cambridge Cambridge UK; ^17^ Division of Molecular and Clinical Medicine University of Dundee Dundee UK; ^18^ Department of Psychology University of Southern Denmark Odense Denmark; ^19^ Department of Medical Psychology Amsterdam University Medical Center, Location Vrije Universiteit Amsterdam the Netherlands; ^20^ Steno Diabetes Center Odense Odense Denmark; ^21^ Department of Diabetes School of Cardiovascular and Metabolic Medicine and Sciences, King's College London London UK

**Keywords:** continuous glucose monitoring (CGM), hypoglycaemia, type 1 diabetes, type 2 diabetes

## Abstract

**Aims:**

Gold and Clarke questionnaires are used to identify impaired awareness of hypoglycaemia (IAH) and severe hypoglycaemic event (SHE) risk in people with diabetes (pwD). We explored their overlap, including Clarke's Hypoglycaemia Awareness Status (Clarke‐HAS) subfactor, and subsection differences in SHE incidence (Gold, Clarke, overlap).

**Materials and Methods:**

This post hoc analysis of the Hypo‐METRICS study recruited pwD on insulin (type 1: T1D; type 2: T2D) with ≥ 1 hypoglycaemic event in the previous 3 months. IAH was defined as Gold ≥ 4, Clarke ≥ 4, Clarke‐HAS ≥ 2.

**Results:**

Gold and Clarke were completed by 232 pwT1D and 285 pwT2D (age: 47 vs. 62 years, *p* < 0.001; HbA1c: 56 mmol/mol (7.3%) vs. 57 mmol/mol (7.4%), *p* = 0.03). IAH prevalence in T1D vs. T2D was 21% vs. 26% (*p* = 0.1), 14% vs. 18% (*p* = 0.2), and 41% vs. 48% (*p* = 0.2) according to Gold, Clarke and Clarke‐HAS. The overlap between Gold ≥ 4, Clarke ≥ 4 was 40% (T1D: 45%; T2D: 37%); between Gold ≥ 4, Clarke‐HAS ≥ 2 it was 45% (T1D: 43%; T2D: 47%). The overlap between Gold ≥ 4, Clarke ≥ 4 was 41% vs. 39% in CGM vs. non‐CGM users (T1D: 50% vs. 35%; T2D: 32% vs. 40%); between Gold ≥ 4, Clarke‐HAS ≥ 2 it was 39% vs. 53% (T1D: 38% vs. 54%; T2D: 40% vs. 53%). In the IAH sample, SHE rate was lower in people found with IAH by Gold vs. Clarke, 9% vs. 53%, and in those assessed by Gold‐Clarke overlap vs. Clarke, 20% vs. 53% (all *p* < 0.001).

**Conclusions:**

Gold and Clarke show moderate consistency (37%–54%) in identifying IAH in insulin‐treated diabetes, with/without CGM use, each associated with different SHE rates, indicating the two questionnaires measure different, independent aspects of IAH.

## 1 Introduction

Hypoglycaemia is an important limiting factor in optimising glycaemic management in diabetes [1], to minimise long‐term complications [2]. Recurrent events can blunt counterregulatory symptom and hormone responses to subsequent events, which in some individuals can lead to impaired awareness of hypoglycaemia (IAH) [3]. IAH, defined as the diminished ability to perceive the onset of hypoglycaemia [4], is associated with increased risk of severe hypoglycaemia in people with type 1 diabetes (pwT1D) [5] and insulin‐treated type 2 diabetes (pwT2D) [6]. IAH is thought to affect up to 25% of pwT1D [5, 7, 8], and about 10% of those with insulin‐treated T2D [6, 9], with evidence from randomised control trials showing that continuous glucose monitoring (CGM) decreases the incidence of severe hypoglycaemia [10].

The Gold measure [11] and the Clarke questionnaire [12] in its updated format [13] were developed in the 1990s as tools for assessing hypoglycaemia awareness in pwT1D. Gold comprises of a single question, with a response option ranging from 1 to 7; Clarke consists of 8 questions, with total score ranging from 0 to 7, with scores ≥ 4 indicating IAH on both measures. Using principal component analysis, in 2020, Sepúlveda and associates highlighted that Clarke incorporates two dimensions: “hypoglycaemia awareness” (questions 1, 2, 5/6, 7, 8) and “severe hypoglycaemia experience” (questions 3 and 4). For the first dimension, the Clarke Hypoglycaemia Awareness Status (Clarke‐HAS) subscale, the authors suggested a score ≥ 2 as indicative of IAH [14]. A later study suggested a value of ≥ 2.5 in pwT2D [15].

Clarke and Gold have been found to highly correlate with one another [16, 17]; both showed a moderately negative relationship with adrenaline responses (Clarke: *r* = −0.51, Gold: *r* = −0.50) and total symptom responses (Clarke: *r* = −0.59, Gold: *r* = −0.57) during clamp‐induced hypoglycaemia in T1D [18]. However, Rubin and associates found that 32% of participants were classified inconsistently by Clarke/Gold questionnaires, although the classification accuracy increased in both extremes of hypoglycaemia awareness [18]. IAH with either measure was associated with lower ratings for autonomic symptoms in daily life in T1D [17]. IAH by either measure is associated with increased severe hypoglycaemia risk [10].

More recently, CGM metrics such as time below range (TBR) are widely used to identify risk of hypoglycaemia but, actually, very few studies link this to SHE risk. Two recently published studies [19, 20] show that high TBR is only associated with increased risk of SHE in the presence of IAH, based on Gold measure. Accurately identifying people with IAH thus remains important for clinical management even with current technology. Given the importance of these questionnaire‐based measures of IAH, we explored whether Gold, Clarke, and Clarke‐HAS measures identify the same individuals with the same awareness status by investigating their overlap and correlations in IAH classification, using data from the Hypo‐METRICS study [21]. Subsequently, severe hypoglycaemia incidence across all IAH classification subgroups was explored.

## 2 Methods

### 2.1 Research Design

This was a post hoc analysis of data collected as part of the Hypo‐METRICS study, a 10‐week multinational observational study (ClinicalTrials.gov (NCT04304963)) conducted at nine clinical sites across Austria, Denmark, France, the Netherlands, and U.K.; data were collected between Oct 2020 and Oct 2022. The study protocol was approved by ethics committees in each country [21]. Written informed consent was obtained from all participants.

### 2.2 Study Participants and Design

Hypo‐METRICS recruited 602 adults aged between 18 and 85 years with T1D and insulin‐treated pwT2D with ≥ 1 hypoglycaemic event in the 3 months prior to study enrolment. Key inclusion criteria were being an adult (aged 18–85 years), living with T1D or T2D, taking ≥ 1 insulin injection per day, and having experienced ≥ 1 episode of hypoglycaemia in the 3 months prior to study enrolment. Key exclusion criteria were an estimated glomerular filtration rate of < 30 mL/min/1.73m^2^ and hybrid‐closed loop use. The trial protocol has been published previously [21].

### 2.3 Measures

Hypoglycaemia awareness status was assessed at enrolment by the Gold question and the Clarke survey. Clarke and Clarke‐HAS scores were calculated by combining the answers to Clarke questions ‘1–8’ and ‘1’, ‘2’ and ‘5–8’, respectively [14]. IAH was defined as Gold ≥ 4, Clarke ≥ 4, and Clarke‐HAS ≥ 2.

### 2.4 Statistical Analysis

Descriptive analysis was used for participants' socio‐demographic and clinical characteristics. Categorical variables were reported as frequencies, percentages and continuous variables as medians and interquartile range to account for the skewness of our data.

We identified proportion of participants with IAH using each measure (Gold, Clarke, Clarke‐HAS) and explored classification differences for the overall sample and by subgroups (diabetes type and mode of glucose monitoring); we additionally calculated their overlap per subgroup, visualising it with Venn diagrams. Agreement was assessed using with Cohen's κ; 95% confidence intervals were derived using Fleiss' method.

We then looked into severe hypoglycaemia incidence differences across all subgroups. Because the Hypo‐METRICS study collected data only for 10 weeks and we did not have prospective documentation of SHE, we used χ^2^ and Fisher's Exact tests to assess the ratio of people with IAH according to each measure and their overlap with ≥ 1 self‐reported event of severe hypoglycaemia the year prior (assessed as response to question 4 of the Clarke survey), subsectioned by modality of glucose monitoring (CGM + SMBG, CGM, and SMBG users), between those with Gold ≥ 4 & Clarke ≤ 3 (*Gold* ≥ *4 only*), Clarke ≥ 4 & Gold ≤ 3 (*Clarke* ≥ *4 only*), and Gold ≥ 4 & Clarke ≥ 4 (*Gold/Clarke overlap*) combined. [Correction added on 26 May 2026, after first online publication: In the previous sentence, ‘question 3’ has been corrected to ‘question 4’.] We extended this analysis to Clarke‐HAS, too. We adjusted with Benjamini‐Hochberg (BH) corrections for multiple comparisons. We used Spearman correlation coefficient (ρ) analysis to investigate the correlations between Gold/Clarke, and Gold/Clarke‐HAS, in the full sample and within each subgroup.

All analyses were performed in R (version 4.4.1) [22]. Statistical significance was defined as *p* ≤ 0.05 (two‐tailed).

## 3 Results

From 602 participants recruited, 517 (232 pwT1D, 285 pwT2D) completed both Gold and Clarke at enrolment (55 did not consent for their data to be used in secondary analyses; 30 had not fully completed all questions for both measures). The list of sociodemographic and clinical characteristics of the sample is in Table 1.

**TABLE 1:** Summary of demographic characteristics of the study sample.

Variable	T1D (*n* = 232)^a^	T2D (*n* = 285)^a^	*p* ^b^
Sex: male, *n* (%)	108 (47)	178 (62)	< 0.001
Age (yr.): median (IQR)	49 (28)	63 (15)	< 0.001
Ethnicity, *n* (%)			0.03
White	223 (96)	260 (91)	
Other ethnicity/did not disclose	9 (4)	25 (9)	
Diabetes duration (yr): median (IQR)	19 (25)	18 (12)	0.3
HbA1c (%), median (IQR)	7.3 (1.1)	7.4 (1.4)	0.03
HbA1c (mmol/mol), median (IQR)	56 (12)	57 (15)	0.03
Unknown	3	0	
Glucose monitoring, *n* (%)			< 0.001
CGM	171 (74)	120 (42)	
SMBG	61 (26)	165 (58)	
Insulin delivery, *n* (%)			< 0.001
Multiple daily injections	148 (64)	148 (52)	
Pump (open loop)	75 (32)	7 (3)	
Other (basal only, mixed insulins)	9 (4)	130 (46)	
Awareness status, *n* (%)			
Gold ≥ 4	48 (21)	75 (26)	0.1
Clarke ≥ 4	32 (14)	51 (18)	0.2
Clarke‐HAS ≥ 2	96 (41)	136 (48)	0.2

^a^ Median (Q1, Q3); *n* (%).

^b^ Wilcoxon rank sum test; Fisher's exact test; χ^2^ test.

### 3.1 Overlap of Gold, Clarke, and Clarke‐HAS in T1D+T2D, T1D, and T2D

The prevalence of IAH in the entire sample was 24% (123/517) by Gold ≥ 4, 16% (83/517) by Clarke ≥ 4, and 45% (232/517) by Clarke‐HAS ≥ 2. There was no difference in the percentage of people identified with IAH between T1D vs. T2D: Gold 21% (48/232) vs. 26% (75/285), *p* = 0.1; Clarke 14% (32/232) vs. 18% (51/285), *p* = 0.2; Clarke‐HAS 41% (96/232) vs. 48% (136/285), *p* = 0.2.

Among pwT1D, (171 CGM, and 61 SMBG users), there was no difference in the percentage of people identified with IAH between CGM vs. SMBG users: Gold 18% (31/171) vs. 28% (17/61), *p* = 0.1; Clarke 15% (26/171) vs. 10% (6/61), *p* = 0.3; Clarke‐HAS 41% (70/171) vs. 43% (26/61), *p* = 0.8. HbA1c was 56 mmol/mol (IQR = 13) vs. 58 mmol/mol (IQR = 11), *p* = 0.4.

Among pwT2D (120 CGM, and 165 SMBG users), there was no difference in the percentage of people identified with IAH between CGM vs. SMBG users: Gold 27% (32/120) vs. 26% (43/165), *p* > 0.9; Clarke 18% (21/120) vs. 18% (30/165), *p* = 0.9. However, a statistically significant difference was found with Clarke‐HAS: 58% (69/120) vs. 41% (67/165) in CGM vs. SMBG‐users, *p* = 0.005. HbA1c was 55 mmol/mol (IQR = 13) vs. 60 mmol/mol (IQR = 19), *p* = 0.008.

**FIGURE 1:**
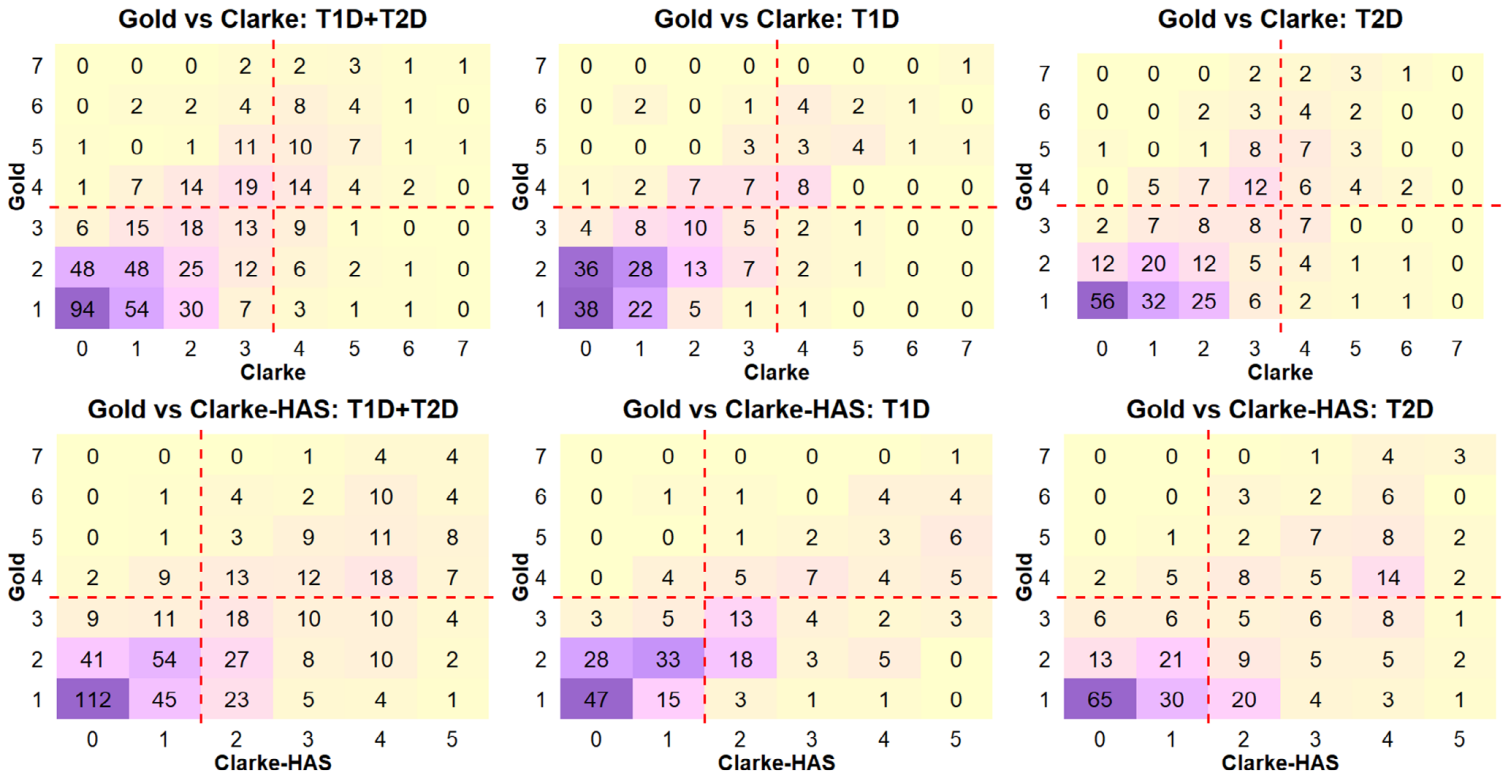
Heatmap matrix demonstrating the number of individuals for each discrete value of Gold, Clarke, and Clarke‐HAS in the entire cohort and diabetes subgroups. The darker the purple shade the higher the count is.

We created a heatmap (Figure 1) that shows how Gold/Clarke, and Gold/Clarke‐HAS map out against each other at each discrete score value in the total sample, T1D, and T2D.

#### 3.1.1 A1: IAH in the Total Sample

**FIGURE 2:**
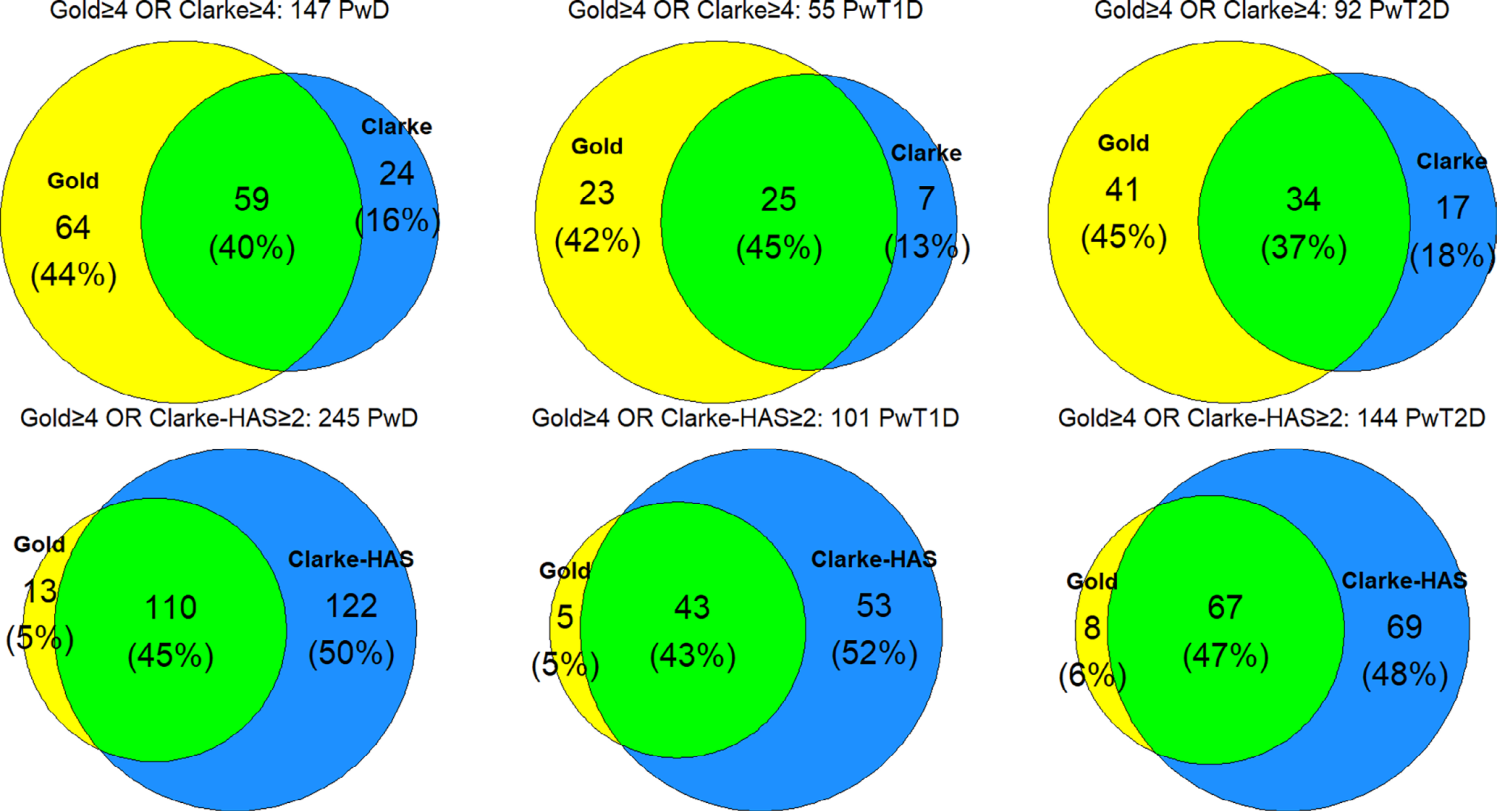
Venn diagram matrix on the overlap between Gold and Clarke, and between Gold and Clarke‐HAS in the entire cohort and diabetes subgroups.

*Gold* ≥ *4 and Clarke* ≥ *4*: Out of 147/517 (28%) participants identified with IAH with either questionnaire, 40% (59/147) were classified with IAH by both measures, leaving 44% (64/147) with IAH by Gold, but aware by Clarke, and 16% (24/147) with IAH by Clarke, but aware by Gold (Figure 2).

*Gold* ≥ *4 and Clarke‐HAS* ≥ *2*: Out of 245/517 (47%) participants identified with IAH with either questionnaire, 45% (110/245) were classified with IAH by both measures, leaving 5% (13/245) with IAH by Gold, but aware by Clarke‐HAS, and 50% (122/245) with IAH by Clarke‐HAS, but aware by Gold.

##### 3.1.1.1 IAH in T1D, T2D

*T1D*: Out of 232 pwT1D, 55 (24%) were identified with IAH with either Gold or Clarke, and 101 (44%) with either Gold or Clarke‐HAS; the overlap of the individuals classified with IAH by both *Gold* and *Clarke* was slightly larger compared to the overlap between both *Gold* and *Clarke‐HAS*, 45% (25/55) and 43% (43/101) respectively.

*T2D*: Out of 285 insulin‐treated pwT2D, 92 (32%) were identified with IAH with either Gold or Clarke, and 144 (51%) with either Gold or Clarke‐HAS; the overlap of the individuals classified with IAH by both *Gold* and *Clarke* was smaller compared to the overlap between both *Gold* and *Clarke‐HAS*, 37% (34/92) and 47% (67/144) respectively.

#### 3.1.2 A2: CGM vs SMBG Users

##### 3.1.2.1 Gold ≥ 4 and Clarke ≥ 4

*CGM*: Out of 291 CGM users, 78 (27%) were identified with IAH by either questionnaire (38 out of 171 (22%) in T1D and 40 out of 120 (33%) in T2D). Of these, 41% (32/78) in T1D + T2D were classified with IAH by both measures, with 50% (19/38) in T1D and 32% (13/40) in T2D (Figure S1).

*SMBG*: Out of 226 SMBG users, 69 (31%) were identified with IAH by either questionnaire (17 out of 61 (28%) in T1D and 52 out of 165 (32%) in T2D). Of these, 39% (27/69) in T1D + T2D were classified with IAH by both measures, with 35% (6/17) in T1D and 40% (21/52) in T2D.

##### 3.1.2.2 Gold ≥ 4 and Clarke‐HAS ≥ 2

*CGM*: Out of 291 CGM users, 145 (50%) were identified with IAH by either questionnaire (73 out of 171 (43%) in T1D and 72 out of 120 (60%) in T2D). Of these, 39% (57/145) in T1D + T2D were classified with IAH by both measures, with 38% (28/73) in T1D and 40% (29/72) in T2D (Figure S2).

*SMBG*: Out of 226 SMBG users, 100 (44%) were identified with IAH by either questionnaire (28 out of 61 (46%) in T1D and 72 out of 165 (44%) in T2D). Of these, 53% (53/100) in T1D + T2D were classified with IAH by both measures, with 54% (15/28) in T1D and 53% (38/72) in T2D.

Observed agreement between Gold and Clarke was moderate overall, between 70%–89%.

Cohen's κ values showed fair‐to‐moderate concordance (≈0.33–0.60), slightly higher in T1D than T2D, and generally stronger for Clarke vs. Clarke‐HAS. The SMBG group often showed equal or higher κ compared with CGM users, especially in Clarke‐HAS comparisons (Table S1).

#### 3.1.3 SHEs in the Year Prior to the Study: IAH and NAH

In the total sample (T1D + T2D, CGM + SMBG), there was no difference in the proportion of SHEs during the 12 months prior to the study between those identified with IAH by *Gold only* (6/64) vs. *Gold and Clarke* (12/59), 9% vs. 20% (*p* = 0.14; BH *p* > 0.9). There was a statistically significant difference between those with IAH by *Gold only* (6/64) vs. *Clarke only* (20/38), 9% vs. 53% respectively (*p* < 0.001; BH *p* < 0.001), and between those with IAH by *Clarke only* vs. *Gold and Clarke*, 53% vs. 20% (*p* < 0.001; BH *p* = 0.01) (Table 2).

**TABLE 2:** Difference in the number and proportion of participants with ≥ 1 severe hypoglycaemic event (SHE) in the year prior to the study within the total sample (T1D + T2D, T1D, and T2D), subsectioned by modality of glucose monitoring (CGM + SMBG, CGM, and SMBG users), between those with Gold ≥ 4 & Clarke ≤ 3 (*Gold ≥ 4 only*), Clarke ≥ 4 & Gold ≤ 3 (*Clarke ≥ 4 only*), and Gold ≥ 4 & Clarke ≥ 4 (*Gold/Clarke overlap*) combined; the same analysis was extended to the redacted version of the Clarke survey (Clarke‐HAS ≥ 2). Benjamini‐Hochberg (BH) correction has been applied for multiple comparisons. If no individuals were identified in an overlapping section, statistical analysis was not possible and results are presented as NA.

Type of diabetes	Mode of glucose monitoring	Awareness status	Proportion (n/N) of participants with ≥ 1 SHE in the year prior to the study	*p*	BH‐adjusted *p*
T1D + T2D	CGM + SMBG	Gold ≥ 4 only vs. Gold/Clarke overlap	9% (6/64) vs. 20% (12/59)	0.14^*a*^	> 0.9
		Gold ≥ 4 only vs. Clarke ≥ 4 only	9% (6/64) vs. 53% (20/38)	< 0.001^*a*^	< 0.001
		Gold/Clarke overlap vs. Clarke ≥ 4 only	20% (12/59) vs. 53% (20/38)	< 0.001^*a*^	0.01
		Gold ≥ 4 only vs. Gold/Clarke‐HAS overlap	8% (1/13) vs. 16% (17/110)	0.69^b^	> 0.9
		Gold ≥ 4 only vs. Clarke‐HAS ≥ 2 only	8% (1/13) vs. 19% (26/136)	0.46^*b*^	0.86
		Gold/Clarke‐HAS overlap vs. Clarke‐HAS ≥ 2 only	16% (17/110) vs. 19% (26/136)	0.56^*a*^	> 0.9
	CGM	Gold ≥ 4 only vs. Gold/Clarke overlap	10% (3/31) vs. 25% (8/32)	0.20^*a*^	0.56
		Gold ≥ 4 only vs. Clarke ≥ 4 only	10% (3/31) vs. 13% (2/15)	> 0.9^*b*^	> 0.9
		Gold/Clarke overlap vs. Clarke ≥ 4 only	25% (8/32) vs. 13% (2/15)	0.47^*b*^	0.86
		Gold ≥ 4 only vs. Gold/Clarke‐HAS overlap	0% (0/6) vs. 19% (11/57)	0.58^*b*^	> 0.9
		Gold ≥ 4 only vs. Clarke‐HAS ≥ 2 only	0% (0/6) vs. 6% (5/82)	> 0.9^*b*^	> 0.9
		Gold/Clarke‐HAS overlap vs. Clarke‐HAS ≥ 2 only	19% (11/57) vs. 6% (5/82)	0.03^*a*^	0.14
	SMBG	Gold ≥ 4 only vs. Gold/Clarke overlap	9% (3/33) vs. 15% (4/27)	0.69^*b*^	> 0.9
		Gold ≥ 4 only vs. Clarke ≥ 4 only	9% (3/33) vs. 78% (18/23)	< 0.001^*a*^	< 0.001
		Gold/Clarke overlap vs. Clarke ≥ 4 only	15% (4/27) vs. 78% (18/23)	< 0.001^*a*^	< 0.001
		Gold ≥ 4 only vs. Gold/Clarke‐HAS overlap	14% (1/7) vs. 11% (6/53)	> 0.9^*b*^	> 0.9
		Gold ≥ 4 only vs. Clarke‐HAS ≥ 2 only	14% (1/7) vs. 39% (21/54)	0.4^*b*^	0.86
		Gold/Clarke‐HAS overlap vs. Clarke‐HAS ≥ 2 only	11% (6/53) vs. 39% (21/54)	< 0.001^*a*^	0.01
T1D	CGM + SMBG	Gold ≥ 4 only vs. Gold/Clarke overlap	13% (3/23) vs. 32% (8/25)	0.22^*a*^	0.58
		Gold ≥ 4 only vs. Clarke ≥ 4 only	13% (3/23) vs. 14% (1/7)	> 0.9^*b*^	> 0.9
		Gold/Clarke overlap vs. Clarke ≥ 4 only	32% (8/25) vs. 14% (1/7)	0.64^*b*^	0.75
		Gold ≥ 4 only vs. Gold/Clarke‐HAS overlap	20% (1/5) vs. 23% (10/43)	> 0.9^*b*^	> 0.9
		Gold ≥ 4 only vs. Clarke‐HAS ≥ 2 only	20% (1/5) vs. 9% (5/53)	0.43^*b*^	0.86
		Gold/Clarke‐HAS overlap vs. Clarke‐HAS ≥ 2 only	23% (10/43) vs. 9% (5/53)	0.12^*a*^	0.4
	CGM	Gold ≥ 4 only vs. Gold/Clarke overlap	8% (1/12) vs. 37% (7/19)	0.11^*b*^	0.4
		Gold ≥ 4 only vs. Clarke ≥ 4 only	8% (1/12) vs. 14% (1/7)	> 0.9^*b*^	> 0.9
		Gold/Clarke overlap vs. Clarke ≥ 4 only	37% (7/19) vs. 14% (1/7)	0.37^*b*^	0.85
		Gold ≥ 4 only vs. Gold/Clarke‐HAS overlap	0% (0/3) vs. 29% (8/28)	0.55^*b*^	> 0.9
		Gold ≥ 4 only vs. Clarke‐HAS ≥ 2 only	0% (0/3) vs. 7% (3/42)	> 0.9^*b*^	> 0.9
		Gold/Clarke‐HAS overlap vs. Clarke‐HAS ≥ 2 only	29% (8/28) vs. 7% (3/42)	0.02^*a*^	0.1
	SMBG	Gold ≥ 4 only vs. Gold/Clarke overlap	18% (2/11) vs. 17% (1/6)	> 0.9	> 0.9
		Gold ≥ 4 only vs. Clarke ≥ 4 only	NA	NA	NA
		Gold/Clarke overlap vs. Clarke ≥ 4 only	NA	NA	NA
		Gold ≥ 4 only vs. Gold/Clarke‐HAS overlap	50% (1/2) vs. 13% (2/15)	0.33^*b*^	0.82
		Gold ≥ 4 only vs. Clarke‐HAS ≥ 2 only	50% (1/2) vs. 18% (2/11)	0.42^*b*^	0.86
		Gold/Clarke‐HAS overlap vs. Clarke‐HAS ≥ 2 only	13% (2/15) vs. 18% (2/11)	> 0.9	> 0.9
T2D	CGM + SMBG	Gold ≥ 4 only vs. Gold/Clarke overlap	7% (3/41) vs. 12% (4/34)	0.69^*b*^	> 0.9
		Gold ≥ 4 only vs. Clarke ≥ 4 only	7% (3/41) vs. 61% (19/31)	< 0.001^*a*^	< 0.001
		Gold/Clarke overlap vs. Clarke ≥ 4 only	12% (4/34) vs. 61% (19/31)	< 0.001^*a*^	< 0.001
		Gold ≥ 4 only vs. Gold/Clarke‐HAS overlap	0% (0/8) vs. 10% (7/67)	> 0.9^*b*^	> 0.9
		Gold ≥ 4 only vs. Clarke‐HAS ≥ 2 only	0% (0/8) vs. 25% (21/83)	0.19^*b*^	0.55
		Gold/Clarke‐HAS overlap vs. Clarke‐HAS ≥ 2 only	10% (7/67) vs. 25% (21/83)	0.03^*a*^	0.14
	CGM	Gold ≥ 4 only vs. Gold/Clarke overlap	11% (2/19) vs. 8% (1/13)	> 0.9^*b*^	> 0.9
		Gold ≥ 4 only vs. Clarke ≥ 4 only	11% (2/19) vs. 13% (1/8)	> 0.9^*b*^	> 0.9
		Gold/Clarke overlap vs. Clarke ≥ 4 only	8% (1/13) vs. 13% (1/8)	> 0.9^*b*^	> 0.9
		Gold ≥ 4 only vs. Gold/Clarke‐HAS overlap	0% (0/3) vs. 10% (3/29)	> 0.9^*b*^	> 0.9
		Gold ≥ 4 only vs. Clarke‐HAS ≥ 2 only	0% (0/3) vs. 5% (2/40)	> 0.9^*b*^	> 0.9
		Gold/Clarke‐HAS overlap vs. Clarke‐HAS ≥ 2 only	10% (3/29) vs. 5% (2/40)	0.64^*b*^	> 0.9
	SMBG	Gold ≥ 4 only vs. Gold/Clarke overlap	5% (1/22) vs. 14% (3/21)	0.34^*b*^	0.82
		Gold ≥ 4 only vs. Clarke ≥ 4 only	5% (1/22) vs. 78% (18/23)	< 0.001^*a*^	< 0.001
		Gold/Clarke overlap vs. Clarke ≥ 4 only	14% (3/21) vs. 78% (18/23)	< 0.001^*a*^	< 0.001
		Gold ≥ 4 only vs. Gold/Clarke‐HAS overlap	0% (0/5) vs. 11% (4/38)	> 0.9^*b*^	> 0.9
		Gold ≥ 4 only vs. Clarke‐HAS ≥ 2 only	0% (0/5) vs. 44% (19/43)	0.14^*b*^	0.44
		Gold/Clarke‐HAS overlap vs. Clarke‐HAS ≥ 2 only	11% (4/38) vs. 44% (19/43)	< 0.001^*a*^	0.01

^a^ Chi‐square test.

^b^ Fisher's exact (Monte Carlo simulation).

##### 3.1.3.1 T1D

In CGM users, *Gold and Clarke‐HAS* identified more people (8/28) with ≥ 1 SHE compared with *Clarke‐HAS only* (3/42), 29% vs. 7%, (*p* = 0.02; BH *p* = 0.1). No other comparison between individual measures or their combinations yielded any statistically significant differences.

In *SMBG* users, we did not find any statistically important differences, although the subgroup sizes were quite small.

##### 3.1.3.2 T2D

Conversely, it was in the *CGM* group that we did not find any statistically important differences, with the subgroup sizes being quite small.

In SMBG users, *Gold only* identified fewer people (1/22) with ≥ 1 SHE compared with *Clarke only* (18/23), 5% vs. 78% (*p* < 0.001; BH *p* < 0.001); similar results were found between *Gold and Clarke* (3/21) vs. *Clarke only* (18/23), 14% vs. 78% (*p* < 0.001; BH *p* < 0.001), and also between *Gold and Clarke‐HAS* (4/38) vs. *Clarke‐HAS only* (19/43), 11% vs. 44% respectively, (*p* < 0.001; BH *p* = 0.01) No other comparison between individual measures or their combinations yielded any statistically significant differences.

For exploratory reasons, we repeated the same analysis using a cutpoint of ≥ 5 for Gold as suggested by the authors of the original paper [11], and ≥ 5 for Clarke as suggested by Matus and associates [23]. We did not find a better discriminatory value among the questionnaires (Table S2).

#### 3.1.4 Correlation Analysis

Table S3 shows the correlation matrices for Gold, Clarke, and Clarke‐HAS continuous scale measures for the total sample, T1D, and T2D.

Between Gold and Clarke, in the entire cohort, the correlation was *ρ* = 0.61 overall, with similar coefficients observed when stratified by diabetes type (0.62 in T1D, 0.63 in T2D; all *p* < 0.001).

Between Gold and Clarke‐HAS, correlation coefficients were similar in magnitude. In the entire cohort, the correlation was *ρ* = 0.65 overall, *ρ* = 0.70 in T1D, and *ρ* = 0.62 in T2D (all *p* < 0.001).

## 4 Discussion

This study highlights considerable discordance between people identified with IAH using widely used assessment tools that are recommended by guidelines such the National Institute for Health and Care Excellence [24] or the American Diabetes Association [25]. These differences are more prominent in CGM users with insulin‐treated T2D.

### 4.1 Discordance of Questionnaires in Identifying IAH

While similar proportion of people were identified as having IAH with different measures, only 40% (45% of T1D and 37% of T2D) were identified as IAH with both Gold and Clarke, with similar figures for the combination of Gold and Clarke‐HAS questionnaires. More people were identified with IAH with just Gold (24%) than Clarke alone (16%). These results are similar to the findings of Rubin and associates, who found 32% of pwT1D were classified inconsistently by Gold/Clarke. Their study (which included 78 pwT1D) also found that these questionnaires poorly predicted hormonal and symptomatic responses to experimental hypoglycaemia [18]. It is worth noting that in the same study, people scoring 3 with either measure were classified as having “indeterminant” awareness status, reducing the prevalence of intact awareness and perhaps contributing to the strength of the correlation. Whilst the authors have computed correlation coefficients to look at agreement between these two measures, we believe that this approach may hide the fact that their overlap is in fact poor: they do not identify the same people with the same awareness status, which is more clinically relevant.

Both Gold and Clarke questionnaires were developed before the CGM era; in our study > 75% of pwT1D and about 30% of pwT2D were CGM users. As most of these participants would have alarms that provide “technological” awareness, this could have affected the interpretation of the Gold question, which asks the individual whether they know when their hypoglycaemic events are commencing. It is possible that some assumed this meant symptoms while others may have included the alerts provided by CGM systems. The Clarke survey is longer and more detailed than the Gold measure, exploring both the ability to detect low glucose as well as frequency of severe and non‐severe hypoglycaemia. This measure is more likely to change with interventions that reduce the frequency of hypoglycaemia such as CGM or HCL, as noted in a recent post hoc analysis of the WISDM study [26].

It was for those reasons that we also looked at Clarke‐HAS as we thought it may align better with Gold. The Clarke‐HAS questionnaire alone identified almost twice as many individuals with IAH in comparison with Gold and almost three times as many when compared with the full version of the Clarke survey, both in T1D and in T2D. To the best of our knowledge, its proposed IAH threshold has not been validated in hypoglycaemic clamp studies, although a recent cross‐sectional observational study found it performed comparably to the Gold questionnaire in predicting severe hypoglycaemia [27].

### 4.2 Awareness Status and Risk of Severe Hypoglycaemia

In its broadest sense, IAH refers to the inability to detect hypoglycaemia, which comes with two conditions. The first is the generation of symptoms associated with hypoglycaemia, related to the hormonal counter‐regulatory response; this is blunted in IAH, described in a hypothesis widely known as hypoglycaemia‐associated autonomic failure (HAAF) [28]; it can only really be assessed with clamp studies. The second condition is the ability of the individual to perceive those symptoms. Whilst in most cases the adrenaline responses and autonomic symptoms are correlated, there can be a discrepancy [29] particularly in those with IAH. Recent data suggest that this relationship may be moderated via interoceptive awareness [30]. Neuroimaging studies suggest this may be mediated via areas in the frontal cortex involved in executive function [31].

CGM and HCL systems have greatly mitigated the risk of SHEs, but residual risk remains as technology can be fallible [8]. Access to CGM systems in different populations may be driven by those individuals experiencing more problematic hypoglycaemia. We also know that over half of sensor‐detected hypoglycaemic episodes are asymptomatic, even in those with normal awareness (as assessed by Gold/Clarke), and those with a low risk of SHEs [32].

Although prevalence of IAH has decreased over the past years [10], recent studies have highlighted the limited predictive value of CGM metrics such as TBR in predicting risk of SHEs [19, 20]. In both of them, people with intact physiological responses had low risk of SHEs, even among those with high TBR; only people with high TBR and IAH combined presented the highest risk. These results render IAH—measured by Gold—an independent predictor of SHEs even in those using advanced technologies.

Most severe hypoglycaemia is concentrated within a small sample of people with recurrent SHEs rather than dispersed across more people with insulin‐treated diabetes [33]. In that regard, asking about the person's past experience of severe hypoglycaemia remains important part of any assessment of future risk of severe hypoglycaemia. Due to the design of the HypoMETRICS study, documentation of SHEs was not prospective and may have been subjected to recall bias; however, SHE rates in our study were not dissimilar to the ones from other real‐world evidence studies [10].

### 4.3 IAH and Person‐Reported Outcomes

Within healthcare systems, awareness status can be a clinical adjunct in the evaluation of people with problematic hypoglycaemia to grant them access to certain therapies, such as HCL systems [34]. IAH status can also negatively impact driving or occupation [35], and is associated with higher levels of diabetes distress [36], depression and anxiety in T1D, even in individuals with access to CGM [37].

### 4.4 IAH: Dichotomy vs Spectrum

The categorisation of intact vs. impaired awareness obscures the fact that IAH is a spectrum rather than a dichotomy [38], which both Gold and Clarke may fail to elucidate when employing a single cut‐off ≥ 4. As demonstrated by the heatmaps in Figure 1, most of the discordance was in the group with “intermediate awareness”, with improved classification accuracy in the extremes [18]. This may suggest a move away from a binary score to a more graduated 3‐level score, similar to the Pedersen‐Bjergaard IAH measure [39] or the DAFNE question [40]. Høi‐Hansen and associates explored this further with a cross‐sectional questionnaire survey which showed that the introduction of an intermediate group in awareness classification, as offered by the Pedersen‐Bjergaard question, corresponded with a third risk profile of severe hypoglycaemia distinct from the other two categories [41]. Of interest, Gold and associates in their landmark paper categorised maintained awareness as 1–2, with IAH 5–7, leaving room for a third category [3, 4], deemed indeterminate [11]. Hypoglycaemia Awareness Questionnaire (Hypo A‐Q), developed on interviews with people living with diabetes and experiences of IAH and the only one so far based on FDA criteria, offers a more nuanced question structure potentially allowing for a more accurate categorisation [42]. A Hypo A‐Q subscale cut‐off score of ≥ 12 indicates impaired awareness [23]. Additionally, even for the purposes of a yes/no assessment, the cut‐offs of ≥ 4 for Gold or Clarke may not be correct, with Matus and associates having found ≥ 5 as the optimal cut‐off for Clarke in clamp‐induced hypoglycaemia [23], whilst, as previously mentioned, a cut‐off of ≥ 2.5 for Clarke‐HAS has been reported effective in pwT2D [15].

We acknowledge the limitations presented as a result of small subgroup sizes when comparing proportion of SHEs at subsections (based on diabetes type, glucose monitoring modality, different IAH measure). For people assessed with intact awareness by any measure, roughly 10% of them reported SHEs, which agrees with rates recently presented in a narrative review about IAH [10]. Those with IAH had a slightly higher rate of SHEs in the year prior irrespective of which measure was used [10]. The reality is that we do not know why some people are identified with IAH with one questionnaire and not with the other. These measures' and their combinations' predictive reliability need to be explored in prospective databases in users of diabetes technology.

## 5 Conclusion

Gold and Clarke questionnaires, widely used to identify people with IAH and risk of severe hypoglycaemia, classify different people as having IAH in approximately 60% of cases. As such, the use of each one in isolation may miss some people at risk of severe hypoglycaemia. The heatmaps suggest that in people with very good awareness or unawareness either measure suffices; the real challenge is in reliably identifying people in the middle of the spectrum. More detailed questionnaires, such as Clarke or Hypo A‐Q may prove more useful in this group. A belt and braces approach may be to use both Gold and Clarke; alternatively, a trichotomous grading of awareness may offer a more granular approach to quantifying risk of hypoglycaemia. While these questionnaires do not measure adrenaline response, they do identify those with reduced perception of hypoglycaemia, and those who have increased SHE risk resulting from higher hypoglycaemia frequency, despite widespread efforts to use technology and metrics such as TBR to stratify risk; this work highlights the need for updating these historical questionnaires for the modern era.

## Author Contributions

V.K. and P.C. developed the plan for the manuscript, with input and advice from the remaining coauthors. V.K. generated the tables and the figures, and prepared the manuscript draft, with critical feedback from the remaining coauthors. V.K. and A.T. conducted the correlation analysis. V.K. conducted the analysis on the proportion of SHEs per IAH group. J.T, G.M.‐E., A.T., P.D., U.S., C.M., S.H., J.K.M., U.P.‐B., B.E.d.G., E.R., M.L.E., R.J.M., J.S., F.P., S.A.A., and P.C. prepared subsequent revisions with feedback from all coauthors. All authors approved the final draft of the manuscript. V.K. is the guarantor of this work and, as such, had full access to all the data in the study and takes responsibility for the integrity of the data and the accuracy of the data analysis.

## Funding

This work was supported by the Innovative Medicines Initiative 2 Joint Undertaking (JU) under grant agreement 777460. JU receives support from the European Union's Horizon 2020 Research and Innovation Programme and European Federation of Pharmaceutical Industries and Associations and T1D Exchange, Juvenile Diabetes Research Foundation, International Diabetes Federation, and The Leona M. and Harry B. Helmsley Charitable Trust. The industry partners supporting JU include Abbott Diabetes Care, Eli Lilly, Medtronic, Novo Nordisk, and Sanofi‐Aventis. Abbott Diabetes Care provided the continuous glucose monitors used in the study. This analysis has been delivered through the National Institute for Health and Care Research (NIHR) Leicester BRC. The views expressed are those of the author(s) and not necessarily those of the National Health Service, NIHR, the Department of Health and Social Care or the JU.

## Conflicts of Interest

V.K. has received funding from KelCon GmbH to attend ATTD 2024. He is also the recipient of YDEF‐Lilly scholarships to attend EASD 2024 and ATTD 2026, and attended the EASD Robert Turner course 2025, partially funded by Lilly. J.J.C.T. has no disclosures to declare. G.E.‐M. has no disclosures to declare. A.T. has no disclosures to declare. P.D. has spoken at educational symposia sponsored by Novonordisk, Grünenthal and Menarini Pharmaceuticals (Ireland) and has served on an advisory board for Dexcom. U.S. has no disclosures to declare. C.L.M. has received research funding and equipment from Dexcom Inc. S.H. undertakes consultancy with Eli Lilly, Novo Nordisk, Zealand Pharma, Vertex, Zucara, Medtronic and receives research support from Dexcom Inc. J.K.M.is a member of advisory boards of Abbott Diabetes Care, Becton‐Dickinson, Biomea Fusion, Dexcom, Eli Lilly, Embecta, Insulet, Medtronic, Novo Nordisk A/S, Pharmasens, Roche Diabetes Care, Sanofi‐Aventis, Tandem. She has received speaker honoraria from A. Menarini Diagnostics, Abbott Diabetes Care, Buzud, Dexcom, Embecta, Eli Lilly, Novo Nordisk A/S, Roche Diabetes Care, Sanofi, Sinocare, Viatris and Ypsomed, She is also a shareholder of decide Clinical Software GmbH and elyte Diagnostics and serves as CMO of elyte Diagnostics. U.P.‐B. has received consulting fees from Abbott, support for attending conferences from Novo Nordisk and Sanofi, and trial supplies from Novo Nordisk. B.E.d.G. has no disclosures to declare. E.R. declares consultant/speaker fees from A. Menarini Diagnostics, Abbott, Air Liquide SI, Astra‐Zeneca, Becton‐Dickinson, Boehringer‐Ingelheim, Cellnovo, Dexcom Inc., Eli‐Lilly, Hillo, Insulet Inc., Johnson & Johnson (Animas, LifeScan), Medtronic, Medirio, Novo‐Nordisk, Roche, and Sanofi‐Aventis and research support by Abbott, Dexcom Inc., Insulet Inc., Roche, and Tandem Diabetes Care. M.L.E. has received personal fees from Abbott Diabetes Care, Eli Lilly and Company, Medtronic, Dexcom, Novo Nordisk, Astra Zeneca, and Zucara. The University of Cambridge has received salary support for M.L.E. from the National Health Service in the East of England through the Clinical Academic Reserve. R.J.M. has no disclosures to declare. J.S. reports honoraria (to research group) from Sanofi and Vertex for service on advisory boards. F.P. has received funding for research from Novo Nordisk, Eli Lilly, and Sanofi‐Aventis Deutschland GmbH. S.A.A. has served on an advisory board for Vertex Pharmaceuticals and has spoken at educational symposia sponsored by Vertex and by Sanofi. No other potential conflicts of interest relevant to this article were reported. P.C. has received personal fees Abbott Diabetes Care, Insulet, Dexcom, Novo Nordisk, AstraZeneca, Medtronic, Roche Diabetes Care, and Sanofi Diabetes and research funding support from Abbott Diabetes Care, Medtronic, and Novo Nordisk.

## Supporting information


**Figure S1:** Venn diagram matrix demonstrating the overlap between Gold and Clarke in CGM vs. SMBG users, in the entire cohort and diabetes subgroups.
**Figure S2:** Venn diagram matrix demonstrating the overlap between Gold and Clarke‐HAS in CGM vs. SMBG users, in the entire cohort and diabetes subgroups.
**Table S1:** Observed agreement and Cohen κ between Gold/Clarke and Gold/Clarke‐HAS within the total sample (T1D + T2D, T1D, and T2D), subsectioned by modality of glucose monitoring (CGM + SMBG, CGM, and SMBG users).
**Table S2:** Difference in the number and proportion of participants with ≥ 1 severe hypoglycemic event (SHE) in the year prior to the study within the total sample (T1D + T2D, T1D, and T2D), subsectioned by modality of glucose monitoring (CGM + SMBG, CGM, and SMBG users), between those with Gold ≥ 5 & Clarke ≤ 4 (*Gold* ≥ *5 only*), Clarke ≥ 5 & Gold ≤ 4 only (*Clarke* ≥ *5 only*), and Gold ≥ 5 & Clarke ≥ 5 (*Gold/Clarke overlap*) combined; the same analysis was extended to the redacted version of the Clarke survey (Clarke‐HAS ≥ 2). Benjamini‐Hochberg (BH) correction has been applied for multiple comparisons. If no individuals were identified in an overlapping section, statistical analysis was not possible and results are presented as NA.
**Table S3:** Spearman's correlation coefficients between Gold and Clarke, and between Gold and Clarke‐HAS.

## Data Availability

The data that support the findings of this study are available from the corresponding author upon reasonable request.

## References

[1] P. E. Cryer , S. N. Davis , and H. Shamoon , “Hypoglycemia in Diabetes,” Diabetes Care 26, no. 6 (2003): 1902–1912, 10.2337/diacare.26.6.1902. 12766131

[2] The Effect of Intensive Treatment of Diabetes on the Development and Progression of Long‐Term Complications in Insulin‐Dependent Diabetes Mellitus,” New England Journal of Medicine 329, no. 14 (1993): 977–986, 10.1056/NEJM199309303291401. 8366922

[3] S. R. Heller and P. E. Cryer , “Reduced Neuroendocrine and Symptomatic Responses to Subsequent Hypoglycemia After 1 Episode of Hypoglycemia in Nondiabetic Humans,” Diabetes 40, no. 2 (1991): 223–226, 10.2337/diab.40.2.223. 1991573

[4] A. J. Graveling and B. M. Frier , “Impaired Awareness of Hypoglycaemia: A Review,” Diabetes & Metabolism 36 (2010): S64–S74, 10.1016/S1262-3636(10)70470-5. 21211739

[5] J. Geddes , J. E. Schopman , N. N. Zammitt , and B. M. Frier , “Prevalence of Impaired Awareness of Hypoglycaemia in Adults With Type 1 Diabetes,” Diabetic Medicine 25, no. 4 (2008): 501–504, 10.1111/j.1464-5491.2008.02413.x. 18387080

[6] L. A. van Meijel , F. de Vegt , E. J. Abbink , et al., “High Prevalence of Impaired Awareness of Hypoglycemia and Severe Hypoglycemia Among People With Insulin‐Treated Type 2 Diabetes: The Dutch Diabetes Pearl Cohort,” BMJ Open Diabetes Research & Care 8, no. 1 (2020): e000935, 10.1136/bmjdrc-2019-000935. PMC7206921 32107264

[7] Y. K. Lin , M. Hung , A. Sharma , et al., “Impaired Awareness of Hypoglycemia Continues to Be a Risk Factor for Severe Hypoglycemia Despite the Use of Continuous Glucose Monitoring System in Type 1 Diabetes,” Endocrine Practice 25, no. 6 (2019): 517–525, 10.4158/EP-2018-0527. 30865520 PMC6771275

[8] J. L. Sherr , L. M. Laffel , J. Liu , et al., “Severe Hypoglycemia and Impaired Awareness of Hypoglycemia Persist in People With Type 1 Diabetes Despite Use of Diabetes Technology: Results From a Cross‐Sectional Survey,” Diabetes Care 47, no. 6 (2024): 941–947, 10.2337/dc23-1765. 38295397 PMC11116910

[9] J. E. Schopman , J. Geddes , and B. M. Frier , “Prevalence of Impaired Awareness of Hypoglycaemia and Frequency of Hypoglycaemia in Insulin‐Treated Type 2 Diabetes,” Diabetes Research and Clinical Practice 87, no. 1 (2010): 64–68, 10.1016/j.diabres.2009.10.013. 19939489

[10] S. A. Berry , A. L. Liarakos , V. Koutroukas , P. Choudhary , E. G. Wilmot , and A. Iqbal , “The Challenge of Assessing Impaired Awareness of Hypoglycaemia in Diabetes in the Era of Continuous Glucose Monitoring: A Narrative Review of Evidence and Translation Into Clinical Practice,” Diabetes, Obesity & Metabolism 27, no. 5 (2025): 2363–2376, 10.1111/dom.16284. PMC11965031 39996361

[11] A. E. Gold , K. M. Macleod , and B. M. Frier , “Frequency of Severe Hypoglycemia in Patients With Type I Diabetes With Impaired Awareness of Hypoglycemia,” Diabetes Care 17, no. 7 (1994): 697–703, 10.2337/diacare.17.7.697. 7924780

[12] W. L. Clarke , D. J. Cox , L. A. Gonder‐Frederick , D. Julian , D. Schlundt , and W. Polonsky , “Reduced Awareness of Hypoglycemia in Adults With IDDM: A Prospective Study of Hypoglycemic Frequency and Associated Symptoms,” Diabetes Care 18, no. 4 (1995): 517–522, 10.2337/diacare.18.4.517. 7497862

[13] S. Little , T. Chadwick , P. Choudhary , et al., “Comparison of Optimised MDI Versus Pumps With or Without Sensors in Severe Hypoglycaemia (The Hypo COMPaSS Trial),” BMC Endocrine Disorders 12, no. 1 (2012): 33, 10.1186/1472-6823-12-33. 23237320 PMC3556156

[14] E. Sepúlveda , R. Poínhos , G. Nata , et al., “Differentiating Hypoglycemia Awareness Status From Hypoglycemia Experience in Tools for Measuring Impaired Awareness of Hypoglycemia,” Diabetes Technology & Therapeutics 22, no. 7 (2020): 541–545, 10.1089/dia.2020.0034. 32175769 PMC7336879

[15] L. C. Ang , Y. M. Bee , S. Y. Goh , and M. M. Teh , “New Insights Into the Currently Available Questionnaire for Assessing Impaired Awareness of Hypoglycaemia (IAH) Among Insulin‐Treated Type 2 Diabetes‐ A Key Risk Factor for Hypoglycaemia,” Diabetes Epidemiology and Management 10 (2023): 100136, 10.1016/j.deman.2023.100136.

[16] K. Ghandi , B. Pieri , A. Dornhorst , and S. Hussain , “A Comparison of Validated Methods Used to Assess Impaired Awareness of Hypoglycaemia in Type 1 Diabetes: An Observational Study,” Diabetes Therapy 12, no. 1 (2021): 441–451, 10.1007/s13300-020-00965-0. 33219468 PMC7843675

[17] J. Geddes , R. J. Wright , N. N. Zammitt , I. J. Deary , and B. M. Frier , “An Evaluation of Methods of Assessing Impaired Awareness of Hypoglycemia in Type 1 Diabetes,” Diabetes Care 30, no. 7 (2007): 1868–1870, 10.2337/dc06-2556. 17416785

[18] N. T. Rubin , E. R. Seaquist , L. Eberly , et al., “Relationship Between Hypoglycemia Awareness Status on Clarke/Gold Methods and Counterregulatory Response to Hypoglycemia,” Journal of the Endocrine Society 6, no. 9 (2022): bvac107, 10.1210/jendso/bvac107. 35935070 PMC9351372

[19] H. Deshmukh , E. G. Wilmot , P. Choudhary , et al., “Time Below Range and Its Influence on Hypoglycemia Awareness and Severe Hypoglycemia: Insights From the Association of British Clinical Diabetologists Study,” Diabetes Care 48, no. 3 (2025): 437–443, 10.2337/dc24-1833. 39746160 PMC11870288

[20] D. Canha , P. Choudhary , E. Cosson , et al., “Time Below Range Alone Is Insufficient to Identify Severe Hypoglycaemia Risk in Type 1 Diabetes—The Critical Role of Hypoglycaemia Awareness: Results From the SFDT1 Study,” Diabetologia 68, no. 12 (2025): 2719–2731, 10.1007/s00125-025-06536-x. 40924111 PMC12594687

[21] P. Divilly , N. Zaremba , Z. Mahmoudi , et al., “Hypo‐METRICS: Hypoglycaemia‐MEasurement, ThResholds and ImpaCtS‐A Multi‐Country Clinical Study to Define the Optimal Threshold and Duration of Sensor‐Detected Hypoglycaemia That Impact the Experience of Hypoglycaemia, Quality of Life and Health Economic Outcomes: The Study Protocol,” Diabetic Medicine 39, no. 9 (2022): e14892, 10.1111/dme.14892. 35633291 PMC9542005

[22] D. Eddelbuettel and R. François , “Rcpp: Seamless *R* and *C++* Integration,” Journal of Statistical Software 40, no. 8 (2011): 1–18, 10.18637/jss.v040.i08.

[23] A. Matus , A. J. Flatt , A. J. Peleckis , C. Dalton‐Bakes , B. Riegel , and M. R. Rickels , “Validating and Establishing a Diagnostic Threshold for the Hypoglycemia Awareness Questionnaire Impaired Awareness Subscale,” Endocrine Practice 29, no. 10 (2023): 762–769, 10.1016/j.eprac.2023.08.004. 37611750 PMC10592063

[24] National Institute for Health and Care Excellence , Type 1 Diabetes in Adults: Diagnosis and Management (Nice Technology Appraisal Guidance TA943, 2023), https://www.nice.org.uk/guidance/ng17.

[25] American Diabetes Association Professional Practice Committee , “6 Glycemic Goals and Hypoglycemia: Standards of Care in Diabetes‐2025,” Diabetes Care 48, no. 1 Suppl 1 (2025): S128–S145, 10.2337/dc25-S006. 39651981 PMC11635034

[26] A. Bilal , F. Yi , K. Whitaker , Z. A. Khan , R. E. Pratley , and A. Casu , “Effects of Continuous Glucose Monitoring on Impaired Awareness of Hypoglycemia in Older Adults With Type 1 Diabetes: A Post Hoc Analysis of the WISDM Study,” Diabetes Care 49, no. 1 (2026): 86–91, 10.2337/dc25-0971. 40902121 PMC12719708

[27] Y. K. Lin , W. Ye , E. Hepworth , et al., “Characterising Impaired Awareness of Hypoglycaemia and Associated Risks Through HypoA‐Q: Findings From a T1D Exchange Cohort,” Diabetologia 68, no. 2 (2025): 433–443, 10.1007/s00125-024-06310-5. 39477881 PMC11837905

[28] S. E. Dagogo‐Jack , S. Craft , and P. E. Cryer , “Hypoglycemia‐Associated Autonomic Failure in Insulin‐Dependent Diabetes Mellitus. Recent Antecedent Hypoglycemia Reduces Autonomic Responses to, Symptoms of, and Defense Against Subsequent Hypoglycemia,” Journal of Clinical Investigation 91 (1993): 819–828. 8450063 10.1172/JCI116302 PMC288033

[29] M. Davis , M. Mellman , S. Friedman , C. J. Chang , and H. Shamoon , “Recovery of Epinephrine Response but Not Hypoglycemic Symptom Threshold After Intensive Therapy in Type 1 Diabetes,” American Journal of Medicine 97, no. 6 (1994): 535–542, 10.1016/0002-9343(94)90349-2. 7985713

[30] A. M. Matus , A. Agni , S. A. Amiel , et al., “Interoceptive Awareness Moderates the Relationship Between Hypoglycemia Exposure and Symptom Recognition in Adults With Type 1 Diabetes,” Diabetes Care 49, no. 3 (2025): 435–443, 10.2337/dc25-2242. PMC12925994 41467850

[31] M. Nwokolo , S. A. Amiel , O. O'Daly , et al., “Impaired Awareness of Hypoglycemia Disrupts Blood Flow to Brain Regions Involved in Arousal and Decision Making in Type 1 Diabetes,” Diabetes Care 42, no. 11 (2019): 2127–2135, 10.2337/dc19-0337. 31455689

[32] P. Divilly , G. Martine‐Edith , N. Zaremba , et al., “Relationship Between Sensor‐Detected Hypoglycemia and Patient‐Reported Hypoglycemia in People With Type 1 and Insulin‐Treated Type 2 Diabetes: The Hypo‐METRICS Study,” Diabetes Care 47, no. 10 (2024): 1769–1777, 10.2337/dc23-2332. 39207738 PMC11417281

[33] U. Pedersen‐Bjergaard , S. Pramming , S. R. Heller , et al., “Severe Hypoglycaemia in 1076 Adult Patients With Type 1 Diabetes: Influence of Risk Markers and Selection,” Diabetes/Metabolism Research and Reviews 20, no. 6 (2004): 479–486, 10.1002/dmrr.482. 15386817

[34] National Institute for Health and Care Excellence , Hybrid Closed Loop Systems for Managing Blood Glucose Levels in Type 1 Diabetes. NICE Technology Appraisal Guidance TA943 (National Institute for Health and Care Excellence, 2023), https://www.nice.org.uk/guidance/ta943. 40245209

[35] GOV.UK , Hypoglycaemia and Driving (UK government, 2025), https://www.gov.uk/hypoglycaemia‐and‐driving.

[36] N. Ali , S. El Hamdaoui , G. Nefs , W. S. JWJ , C. J. Tack , and B. E. de Galan , “High Diabetes‐Specific Distress Among Adults With Type 1 Diabetes and Impaired Awareness of Hypoglycaemia Despite Widespread Use of Sensor Technology,” Diabetic Medicine 40, no. 9 (2023): e15167, 10.1111/dme.15167. 37347681

[37] Y. K. Lin , E. Hepworth , N. de Zoysa , et al., “Relationships of Hypoglycemia Awareness, Hypoglycemia Beliefs, and Continuous Glucose Monitoring Glycemic Profiles With Anxiety and Depression Symptoms in Adults With Type 1 Diabetes Using Continuous Glucose Monitoring Systems,” Diabetes Research and Clinical Practice 209 (2024): 111596, 10.1016/j.diabres.2024.111596. 38428746 PMC10960959

[38] B. M. Frier , “Impaired Awareness of Hypoglycaemia,” in Hypoglycaemia in Clinical Diabetes, 1st ed., ed. B. M. Frier , S. R. Heller , and R. J. McCrimmon (Wiley, 2014), 114–144, 10.1002/9781118695432.ch6.

[39] U. Pedersen‐Bjergaard , S. Pramming , and B. Thorsteinsson , “Recall of Severe Hypoglycaemia and Self‐Estimated State of Awareness in Type 1 Diabetes,” Diabetes/Metabolism Research and Reviews 19, no. 3 (2003): 232–240, 10.1002/dmrr.377. 12789657

[40] N. de Zoysa , H. Rogers , M. Stadler , et al., “A Psychoeducational Program to Restore Hypoglycemia Awareness: The DAFNE‐HART Pilot Study,” Diabetes Care 37, no. 3 (2014): 863–866, 10.2337/dc13-1245. 24319119

[41] T. Høi‐Hansen , U. Pedersen‐Bjergaard , and B. Thorsteinsson , “Classification of Hypoglycemia Awareness in People With Type 1 Diabetes in Clinical Practice,” Journal of Diabetes and Its Complications 24, no. 6 (2010): 392–397, 10.1016/j.jdiacomp.2009.07.006. 19796968

[42] J. Speight , S. M. Barendse , H. Singh , et al., “Characterizing Problematic Hypoglycaemia: Iterative Design and Preliminary Psychometric Validation of the Hypoglycaemia Awareness Questionnaire (HypoA‐Q),” Diabetic Medicine 33, no. 3 (2016): 376–385, 10.1111/dme.12824. 26042777

