# Supplementary material for: Gold and Clarke Questionnaires Identify Different People With Insulin‐Treated Diabetes and Impaired Awareness of Hypoglycaemia in Almost 60% of Cases: A Post Hoc Analysis From the Hypo‐METRICS Study
Source: Diabetes Obes Metab. 2026 May 11;28(8):6755–66. doi: 10.1111/dom.70869 (PMC13341389; doi:10.1111/dom.70869)
Supplement: Supplementary file 1 — Figure S1: Venn diagram matrix demonstrating the overlap between Gold and Clarke in CGM vs. SMBG users, in the entire cohort and diabetes subgroups. Figure S2: Venn diagram matrix demonstrating the overlap between Gold and Clarke‐HAS in CGM vs. SMBG users, in the entire cohort and diabetes subgroups. Table S1: Observed agreement and Cohen κ between Gold/Clarke and Gold/Clarke‐HAS within the total sample (T1D + T2D, T1D, and T2D), subsectioned by modality of glucose monitoring (CGM + SMBG, CGM, and SMBG users). Table S2: Difference in the number and proportion of participants with ≥ 1 severe hypoglycemic event (SHE) in the year prior to the study within the total sample (T1D + T2D, T1D, and T2D), subsectioned by modality of glucose monitoring (CGM + SMBG, CGM, and SMBG users), between those with Gold ≥ 5 & Clarke ≤ 4 (Gold ≥ 5 only), Clarke ≥ 5 & Gold ≤ 4 only (Clarke ≥ 5 only), and Gold ≥ 5 & Clarke ≥ 5 (Gold/Clarke overlap) combined; the same analysis was extended to the redacted version of the Clarke survey (Clarke‐HAS ≥ 2). Benjamini‐Hochberg (BH) correction has been applied for multiple comparisons. If no individuals were identified in an overlapping section, statistical analysis was not possible and results are presented as NA. Table S3: Spearman's correlation coefficients between Gold and Clarke, and between Gold and Clarke‐HAS. [file DOM-28-6755-s001.docx]

**Supplemental material
Supplementary figure 1:** Venn diagram matrix demonstrating the overlap between Gold and Clarke in CGM vs SMBG users, in the entire cohort and diabetes subgroups.

| **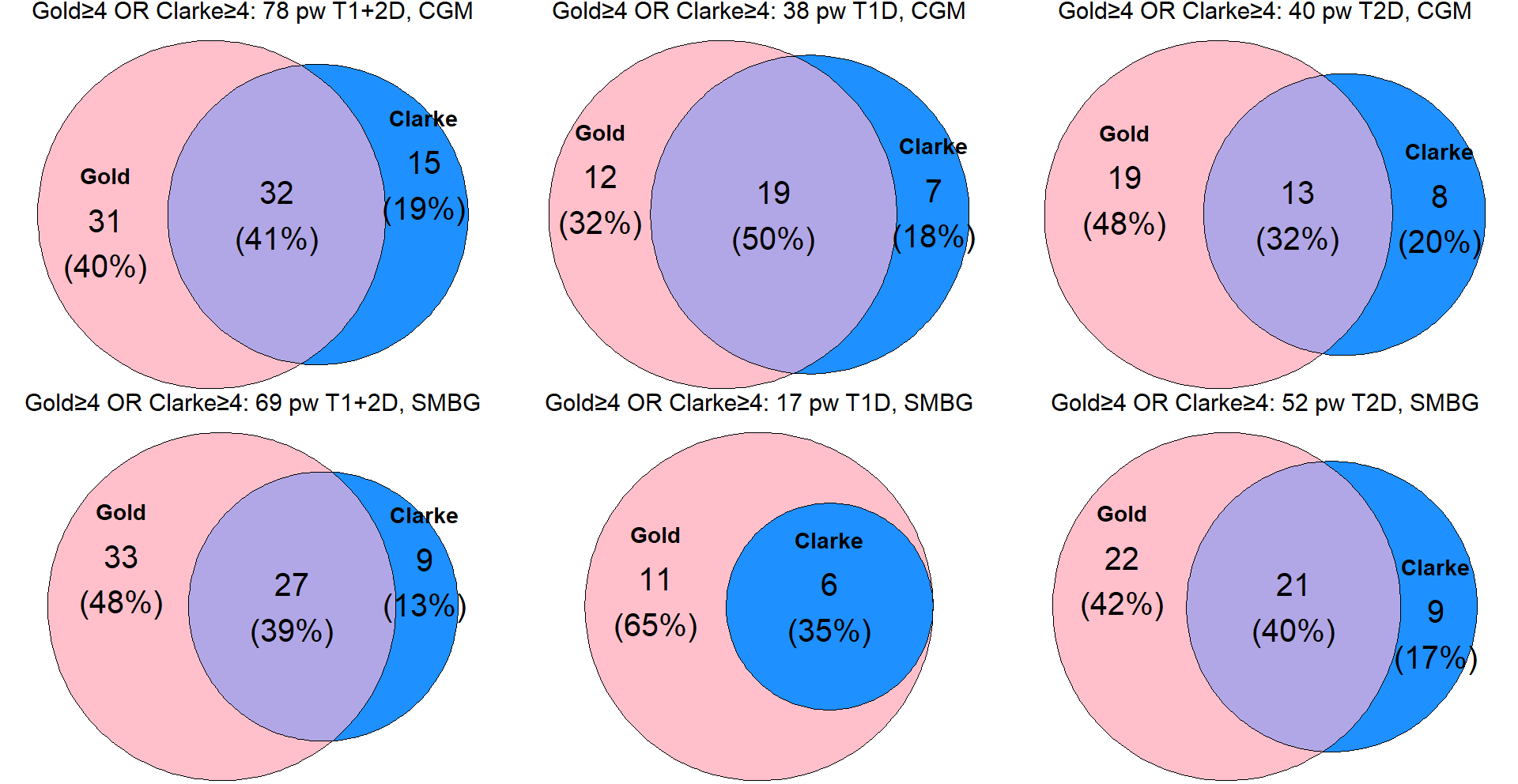** |
| --- |

**Supplementary figure 2:** Venn diagram matrix demonstrating the overlap between Gold and Clarke-HAS in CGM vs SMBG users, in the entire cohort and diabetes subgroups.

| **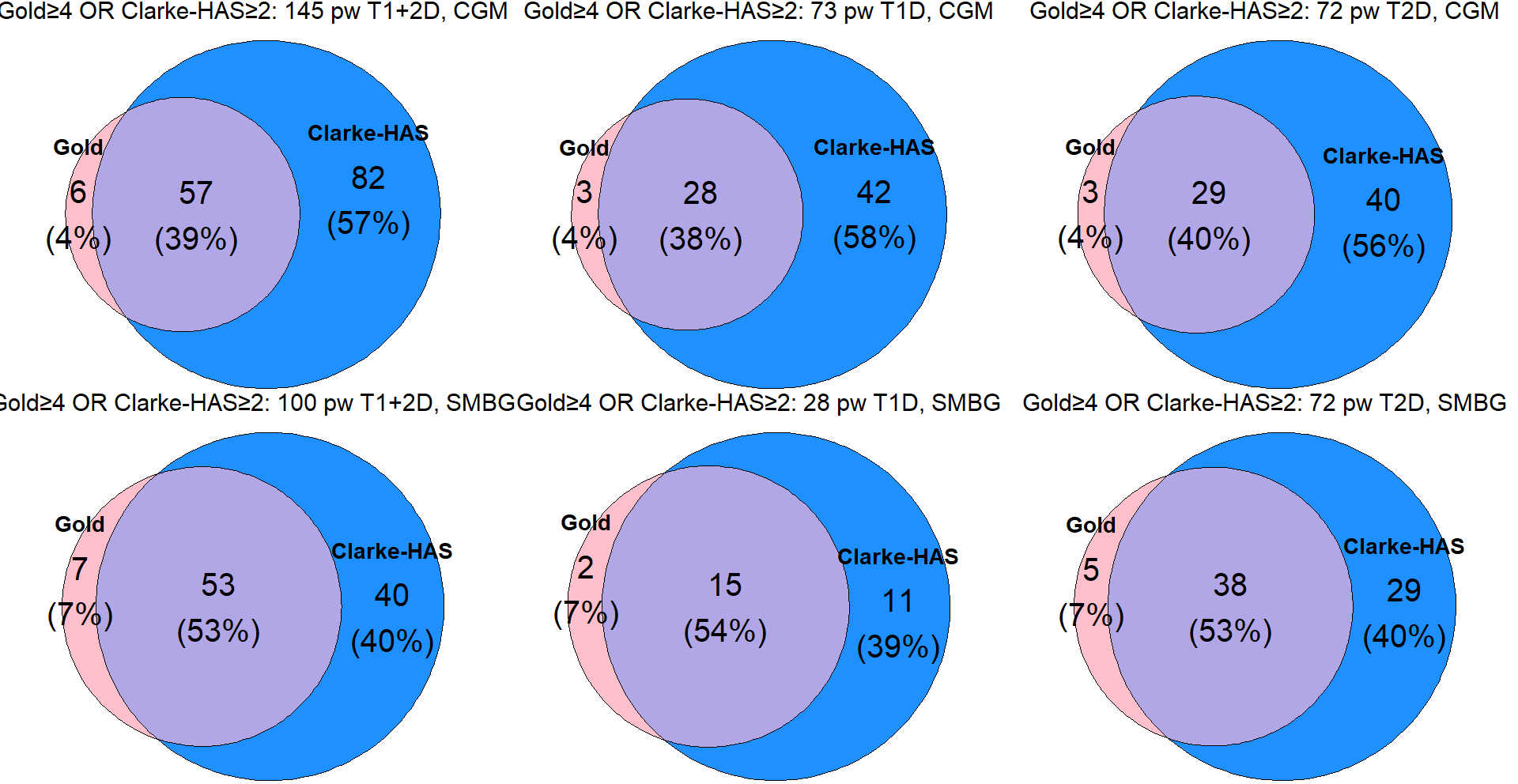** |
| --- |

**S**upplementary table 1: Observed agreement and Cohen κ between Gold/Clarke and Gold/Clarke-HAS within the total sample (T1D+T2D, T1D, and T2D), subsectioned by modality of glucose monitoring (CGM+SMBG, CGM, and SMBG users).

| **Type of diabetes** | **Mode of glucose monitoring** | **Overlap comparison** | **Observed agreement (%)** | **Cohen's κ (95% CI)** |
| --- | --- | --- | --- | --- |
| T1D+T2D | CGM+SMBG | Gold vs Clarke | 83 | 0.47 (0.37–0.56) |
| T1D+T2D | CGM | Gold vs Clarke | 84.2 | 0.49 (0.35–0.60) |
| T1D+T2D | SMBG | Gold vs Clarke | 81.4 | 0.45 (0.31–0.58) |
| T1D+T2D | CGM+SMBG | Gold vs Clarke-HAS | 73.9 | 0.45 (0.26–0.64) |
| T1D+T2D | CGM | Gold vs Clarke-HAS | 69.8 | 0.38 (0.13–0.63) |
| T1D+T2D | SMBG | Gold vs Clarke-HAS | 79.2 | 0.55 (0.25–0.84) |
| T1D | CGM+SMBG | Gold vs Clarke | 87.1 | 0.55 (0.40–0.69) |
| T1D | CGM | Gold vs Clarke | 88.9 | 0.60 (0.42–0.76) |
| T1D | SMBG | Gold vs Clarke | 82 | 0.44 (0.19–0.69) |
| T1D | CGM+SMBG | Gold vs Clarke-HAS | 75 | 0.44 (0.17–0.72) |
| T1D | CGM | Gold vs Clarke-HAS | 73.7 | 0.40 (0.10–0.71) |
| T1D | SMBG | Gold vs Clarke-HAS | 78.7 | 0.54 (-0.03–1.12) |
| T2D | CGM+SMBG | Gold vs Clarke | 79.6 | 0.42 (0.29–0.53) |
| T2D | CGM | Gold vs Clarke | 77.5 | 0.35 (0.15–0.54) |
| T2D | SMBG | Gold vs Clarke | 81.2 | 0.46 (0.29–0.61) |
| T2D | CGM+SMBG | Gold vs Clarke-HAS | 73 | 0.45 (0.19–0.71) |
| T2D | CGM | Gold vs Clarke-HAS | 64.2 | 0.33 (-0.04–0.70) |
| T2D | SMBG | Gold vs Clarke-HAS | 79.4 | 0.55 (0.20–0.89) |

**Supplementary table 2**: Difference in the number and proportion of participants with ≥1 severe hypoglycemic event (SHE) in the year prior to the study within the total sample (T1D+T2D, T1D, and T2D), subsectioned by modality of glucose monitoring (CGM+SMBG, CGM, and SMBG users), between those with Gold≥5 & Clarke≤4 (*Gold≥5 only*), Clarke≥5 & Gold≤4 only (*Clarke≥5 only*), and Gold≥5 & Clarke≥5 (*Gold/Clarke overlap*) combined; the same analysis was extended to the redacted version of the Clarke survey (Clarke-HAS≥2). Benjamini-Hochberg (BH) correction has been applied for multiple comparisons. If no individuals were identified in an overlapping section, statistical analysis was not possible and results are presented as NA.

| **Type of diabetes** | **Mode of glucose monitoring** | **Awareness status** | **Proportion (n/N) of participants with ≥1 SHE in the year prior to the study** | **p-value** | **BH-adjusted p-value** |
| --- | --- | --- | --- | --- | --- |
| T1D+T2D | CGM+SMBG | Gold≥5 only vs Gold/Clarke overlap | **5%** (2/43) vs **37%** (7/19) | **<0.001*^2^*** | 0.06 |
|  |  | Gold≥5 only vs Clarke≥5 only | **5%** (2/43) vs **33%** (4/12) | **0.02*^2^*** | 0.13 |
|  |  | Gold/Clarke overlap vs Clarke≥5 only | **37%** (7/19) vs **33%** (4/12) | >0.9^2^ | >0.9 |
|  |  | Gold≥5 only vs Gold/Clarke-HAS overlap | **0%** (0/2) vs **15%** (9/60) | >0.9*^2^* | >0.9 |
|  |  | Gold≥5 only vs Clarke-HAS≥2 only | **0%** (0/2) vs **18%** (34/186) | >0.9*^2^* | >0.9 |
|  |  | Gold/Clarke-HAS overlap vs Clarke-HAS≥2 only | **15%** (9/60) vs **18%** (34/186) | 0.7*^1^* | >0.9 |
|  | CGM | Gold≥5 only vs Gold/Clarke overlap | **0%** (0/18) vs **46%** (6/13) | **<0.001*^2^*** | 0.06 |
|  |  | Gold≥5 only vs Clarke≥5 only | **0%** (0/18) vs **0%** (0/6) | >0.9*^2^* | >0.9 |
|  |  | Gold/Clarke overlap vs Clarke≥5 only | **46%** (6/13) vs **0%** (0/6) | 0.11^2^ | 0.36 |
|  |  | Gold≥5 only vs Gold/Clarke-HAS overlap | **0%** (0/1) vs **20%** (6/30) | >0.9*^2^* | >0.9 |
|  |  | Gold≥5 & Clarke-HAS≤1 vs Clarke-HAS≥2 only | **0%** (0/1) vs **9%** (10/109) | >0.9*^2^* | >0.9 |
|  |  | Gold/Clarke-HAS overlap vs Clarke-HAS≥2 only | **20%** (6/30) vs **9%** (10/109) | **0.11*^2^*** | 0.11 |
|  | SMBG | Gold≥5 only vs Gold/Clarke overlap | **8%** (2/25) vs **17%** (1/6) | 0.49*^2^* | >0.9 |
|  |  | Gold≥5 only vs Clarke≥5 only | **8%** (2/25) vs **67%** (4/6) | **0.01*^2^*** | 0.08 |
|  |  | Gold/Clarke overlap vs Clarke≥5 only | **17%** (1/6) vs **67%** (4/6) | 0.24*^2^* | 0.65 |
|  |  | Gold≥5 only vs Gold/Clarke-HAS overlap | **0%** (0/1) vs **10%** (3/30) | >0.9*^2^* | >0.9 |
|  |  | Gold≥5 only vs Clarke-HAS≥2 only | **0%** (0/1) vs **31%** (24/77) | >0.9*^2^* | >0.9 |
|  |  | Gold/Clarke-HAS overlap vs Clarke-HAS≥2 only | **10%** (3/30) vs **31%** (24/77) | **0.04*^1^*** | 0.23 |
| T1D | CGM+SMBG | Gold≥5 only vs Gold/Clarke overlap | **8%** (1/13) vs **50%** (5/10) | **0.05*^2^*** | 0.25 |
|  |  | Gold≥5 only vs Clarke≥5 only | **8%** (1/13) vs **0%** (0/2) | >0.9*^2^* | >0.9 |
|  |  | Gold/Clarke overlap vs Clarke≥5 only | **50%** (5/10) vs **0%** (0/2) | 0.47*^2^* | >0.9 |
|  |  | Gold≥5 only vs Gold/Clarke-HAS overlap | **0%** (0/1) vs **27%** (6/22) | >0.9*^2^* | >0.9 |
|  |  | Gold≥5 only vs Clarke-HAS≥2 only | **0%** (0/1) vs **12%** (9/74) | >0.9*^2^* | >0.9 |
|  |  | Gold/Clarke-HAS overlap vs Clarke-HAS≥2 only | **27%** (6/22) vs **12%** (9/74) | 0.1*^2^* | 0.36 |
|  | CGM | Gold≥5 only vs Gold/Clarke overlap | **0%** (0/8) vs **56%** (5/9) | **0.03^2^** | 0.18 |
|  |  | Gold≥5 only vs Clarke≥5 only | **0%** (0/8) vs **0%** (0/2) | >0.9*^2^* | >0.9 |
|  |  | Gold/Clarke overlap vs Clarke≥5 only | **56%** (5/9) vs **0%** (0/2) | 0.45*^2^* | >0.9 |
|  |  | Gold≥5 only vs Gold/Clarke-HAS overlap | **0%** (0/1) vs **31%** (5/16) | >0.9*^2^* | >0.9 |
|  |  | Gold≥5 only vs Clarke-HAS≥2 only | **0%** (0/1) vs **11%** (6/54) | >0.9*^2^* | >0.9 |
|  |  | Gold/Clarke-HAS overlap vs Clarke-HAS≥2 only | **31%** (5/16) vs **11%** (6/54) | 0.11*^2^* | 0.36 |
|  | SMBG | Gold≥5 only vs Gold/Clarke overlap | **20%** (1/5) vs **0%** (0/1) | >0.9*^2^* | >0.9 |
|  |  | Gold≥5 only vs Clarke≥5 only | NA | NA | NA |
|  |  | Gold/Clarke overlap vs Clarke≥5 only | NA | NA | NA |
|  |  | Gold≥5 only vs Gold/Clarke-HAS overlap | NA | NA | NA |
|  |  | Gold≥5 only vs Clarke-HAS≥2 only | NA | NA | NA |
|  |  | Gold/Clarke-HAS overlap vs Clarke-HAS≥2 only | **17%** (1/6) vs **15%** (3/20) | >0.9*^2^* | >0.9 |
| T2D | CGM+SMBG | Gold≥5 only vs Gold/Clarke overlap | **3%** (1/30) vs **22%** (2/9) | 0.13*^2^* | 0.38 |
|  |  | Gold≥5 only vs Clarke≥5 only | **3%** (1/30) vs **40%** (4/10) | **0.01*^2^*** | 0.1 |
|  |  | Gold/Clarke overlap vs Clarke≥5 only | **22%** (2/9) vs **40%** (4/10) | 0.63*^2^* | >0.9 |
|  |  | Gold≥5 only vs Gold/Clarke-HAS overlap | **0%** (0/1) vs **8%** (3/38) | >0.9*^2^* | >0.9 |
|  |  | Gold≥5 only vs Clarke-HAS≥2 only | **0%** (0/1) vs **22%** (25/112) | >0.9*^2^* | >0.9 |
|  |  | Gold/Clarke-HAS overlap vs Clarke-HAS≥2 only | **8%** (3/38) vs **22%** (25/112) | 0.08^1^ | 0.36 |
|  | CGM | Gold≥5 only vs Gold/Clarke overlap | **0%** (0/10) vs **25%** (1/4) | 0.29*^2^* | 0.72 |
|  |  | Gold≥5 only vs Clarke≥5 only | **0%** (0/10) vs **0%** (0/4) | >0.9*^2^* | >0.9 |
|  |  | Gold/Clarke overlap vs Clarke≥5 only | **25%** (1/4) vs **0%** (0/4) | >0.9*^2^* | >0.9 |
|  |  | Gold≥5 only vs Gold/Clarke-HAS overlap | NA | NA | NA |
|  |  | Gold≥5 only vs Clarke-HAS≥2 only | NA | NA | NA |
|  |  | Gold/Clarke-HAS overlap vs Clarke-HAS≥2 only | **7%** (1/14) vs **7%** (4/55) | >0.9*^2^* | >0.9 |
|  | SMBG | Gold≥5 only vs Gold/Clarke overlap | **5%** (1/20) vs **20%** (1/5) | 0.37*^2^* | 0.88 |
|  |  | Gold≥5 only vs Clarke≥5 only | **5%** (1/20) vs **67%** (4/6) | **<0.001*^2^*** | 0.07 |
|  |  | Gold/Clarke overlap vs Clarke≥5 only | **20%** (1/5) vs **67%** (4/6) | 0.24*^2^* | 0.65 |
|  |  | Gold≥5 only vs Gold/Clarke-HAS overlap | **0%** (0/1) vs **8%** (2/24) | >0.9*^2^* | >0.9 |
|  |  | Gold≥5 only vs Clarke-HAS≥2 only | **0%** (0/1) vs **37%** (21/57) | >0.9*^2^* | >0.9 |
|  |  | Gold/Clarke-HAS overlap vs Clarke-HAS≥2 only | **8%** (2/24) vs **37%** (21/57) | **0.02*^1^*** | 0.14 |
| *^1^* Chi-square test |  |  |  |  |  |
| *^2^* Fisher’s Exact (Monte Carlo simulation) |  |  |  |  |  |

**Supplementary t**able 3: Spearman’s correlation coefficients between Gold and Clarke, and between Gold and Clarke-HAS.

| **Pair of questionnaires** | **Glucose monitoring mode** | **Diabetes type** | **Spearman ρ co-efficient** | **p-value** |
| --- | --- | --- | --- | --- |
| Gold, Clarke | CGM+SMBG | T1D+T2D | 0.61 | <0.001 |
|  |  | T1D | 0.62 | <0.001 |
|  |  | T2D | 0.63 | <0.001 |
|  | CGM | T1D+T2D | 0.57 | <0.001 |
|  |  | T1D | 0.55 | <0.001 |
|  |  | T2D | 0.60 | <0.001 |
|  | SMBG | T1D+T2D | 0.66 | <0.001 |
|  |  | T1D | 0.78 | <0.001 |
|  |  | T2D | 0.64 | <0.001 |
| Gold, Clarke-HAS | CGM+SMBG | T1D+T2D | 0.65 | <0.001 |
|  |  | T1D | 0.70 | <0.001 |
|  |  | T2D | 0.62 | <0.001 |
|  | CGM | T1D+T2D | 0.62 | <0.001 |
|  |  | T1D | 0.65 | <0.001 |
|  |  | T2D | 0.59 | <0.001 |
|  | SMBG | T1D+T2D | 0.68 | <0.001 |
|  |  | T1D | 0.84 | <0.001 |
|  |  | T2D | 0.63 | <0.001 |
| *^1^* Chi-square test |  |  |  |  |
| *^2^* Fisher’s Exact (Monte Carlo simulation) |  |  |  |  |
